# Carvacrol and Streptomycin in Combination Weaken Streptomycin Resistance in *Pectobacterium carotovorum* subsp. *carotovorum*

**DOI:** 10.3390/plants14060908

**Published:** 2025-03-14

**Authors:** Yue Shen, Yiying Li, Litao Wang, Chenying Wu, Xu Su, Yongqiang Tian

**Affiliations:** 1 School of Biological and Pharmaceutical Engineering, Lanzhou Jiaotong University, Lanzhou 730070, China; 2 Department of Biological Sciences, School of Science, Xi’an Jiaotong-Liverpool University, Suzhou 215123, China; 3 Key Laboratory of Biodiversity Formation Mechanism and Comprehensive Utilization of the Qinghai-Tibet Plateau in Qinghai Province, Qinghai Normal University, Xining 810008, China

**Keywords:** carvacrol, *Pectobacterium carotovorum* subsp. *carotovorum*, soft rot, streptomycin resistance

## Abstract

*Pectobacterium carotovorum* subsp. *carotovorum* (*Pcc*) is a major phytopathogen responsible for soft rot in vegetables, affecting various staple crops such as carrots and potatoes. However, the recent emergence of streptomycin-resistant strains of *Pcc* has compromised the effectiveness of streptomycin for treating disease in agriculture. This study aimed to evaluate the effects of the phenolic compounds carvacrol, streptomycin, and a combination of both on the antibacterial activity, cell membrane integrity, and virulence factors of a streptomycin-resistant strain of *Pcc* (SP). The results revealed that the minimum inhibitory concentrations (MIC) of carvacrol and streptomycin against the SP strain were 200 μL/L and 50 g/L, respectively. In particular, their combined application had an additive effect on SP (fractional inhibitory concentration index, FICI = 0.625), leading to 2-fold and 8-fold reductions in the concentrations of the combined use of carvacrol and streptomycin, respectively, compared to when used alone. Follow-up control tests using detached Chinese cabbage, potato, and carrot samples showed that the combined treatment significantly alleviates the severity of soft rot disease and inhibits the relative conductivity, motility, and extracellular hydrolase secretion of SP. The scanning electron microscopy and confocal laser scanning microscopy observations further confirmed the disruption of SP’s cell membrane permeability and cell wall integrity after treatment with both carvacrol and streptomycin. Additionally, the transcriptome analysis indicated that their combined use enhanced the suppression of SP by regulating genes associated with its membrane integrity, virulence factors, and resistance mechanisms. In conclusion, applying the phenol–antibiotic combination of carvacrol and streptomycin significantly reduces the streptomycin dose needed against SP and can effectively control soft rot in vegetables prone to it, offering a potential management strategy for controlling SP-induced soft rot during postharvest storage.

## 1. Introduction

Soft rot, caused primarily by *Pectobacterium carotovorum* subsp. *carotovorum* (*Pcc*), ranks among the most devastating bacterial diseases affecting plants globally [1]. *Pcc* is a Gram-negative bacterium that can produce an array of plant cell wall-degrading enzymes—including cellulase, polygalacturonase, pectate lyase, xylanase, and protease—which lead to cell necrosis or tissue impregnation in plants, resulting in various types of soft rot disease, such as those impacting carrot, potato, and broccoli crops [2,3,4,5]. In the past decade, the global agricultural sector has incurred substantial economic losses due to the postharvest deterioration of produce and their chemical cross-contamination, both of which are linked to *Pcc* [6,7]. In addition, this pathogen is distributed worldwide, being highly infectious and persistent, capable of surviving as a parasite in soil or plant tissue for over a year and spreading to nearby crops [8]. Hence, *Pcc* is deemed one of the top ten critical phytopathogens in modern agriculture [9].

To prevent and control soft rot in vegetables, the prevailing management approach still relies on chemical methods, with synthetic insecticides and agricultural antibiotics frequently applied [10]. Among these, one of the most widely applied antibiotics for soft rot control is streptomycin [11,12], an aminoglycoside antibiotic produced by *Streptomyces griseus* [13] that inhibits protein synthesis and has been used to control plant bacterial diseases since the early 1950s [14]. Soft rot disease is often managed with antibiotics, but their excessive use can drive the evolution of resistance in causal bacteria, leading to less efficient control, the pollution of ecosystems, and potential human health concerns, including cancer risks [15,16,17]. Accordingly, the problem of streptomycin resistance has now caught the world’s attention [18]; for instance, resistance to streptomycin has been found in major crop pathogens such as *Erwinia amylovora* [19] and *P. syringae* pv. *syringae* [20]. Moreover, recent findings by Kim et al. indicate that the minimum inhibitory concentration (MIC) of streptomycin against *Pectobacterium* strains isolated in 2021 was double that of earlier strains [21]. This highlights the pressing necessity for innovative and efficient strategies to better manage soft rot in agricultural production.

Recently, increasing attention has shifted to natural antimicrobial agents due to their promising potential for controlling multidrug-resistant pathogens [22,23,24,25]. The phenolic compound carvacrol, whose chemical formula is 2-Methyl-5-(propan-2-yl)phenol, belongs to the monoterpenoid phenols found in essential oils obtained from oregano, thyme, and various plant species in the Lamiaceae family [26,27,28,29] that possess strong and broad-spectrum antibacterial activities [30,31,32]. Notably, carvacrol also inhibits biofilm formation in foodborne pathogens and spoilage-causing bacteria. Its principal antibacterial mechanism involves disrupting the cytoplasmic membrane, leading to greater permeability and membrane depolarization [33,34,35]. Furthermore, carvacrol seems capable of inhibiting ATP synthesis, thereby affecting energy-dependent processes, such as enzyme activity and botulinum toxin production [36]. Most importantly, we know that essential oils can enhance an antibiotic’s efficacy when used in combination with it, either by desensitizing isolates that are resistant to the antibiotic [37] or by reducing the dose or toxicity of certain antibiotics [38,39], resulting in more potent antimicrobial activity than when a given antibiotic (or essential oil) is used alone. For example, carvacrol combined with meropenem has a synergistic effect on the inhibition of carbapenem-resistant *Klebsiella pneumoniae* [40], and carvacrol and cefixime together confer enhanced antibacterial and antibiofilm properties [41]. Moreover, tested combinations of phytotannins and antibiotics have demonstrated synergistic effects against both resistant and susceptible strains of *Acinetobacter baumannii* [42].

Therefore, in the context of mitigating soft rot, this study investigated the impacts of a combined carvacrol and streptomycin treatment on streptomycin-resistant *Pcc*, using physiological and biochemical experiments such as the time–kill curve, relative conductivity, motility, SEM, CLSM, and extracellular hydrolase secretion, and revealed the effects of carvacrol combined with streptomycin on SP cell wall, membrane, and virulence factors. We also carried out a transcriptome analysis to uncover the synergistic antibacterial mechanism operating at the molecular level between carvacrol and streptomycin vis-à-vis SP. This empirical study offers a foundation for developing disease control technology to address postharvest soft rot in streptomycin-resistant SP crops.

## 2. Results

### 2.1. MICs of Carvacrol, Streptomycin, and Their Combination Against SP

In this study, one strain of streptomycin-resistant *Pcc* was continuously subcultured in a stepwise manner through increasing concentrations of streptomycin. At the onset of this process, the initial strain was exposed to 20 mg/L of streptomycin, whose concentration was doubled every 3 days thereafter. Meanwhile, the MIC of *Pcc* increased significantly, such that after 45 days of continual subculturing, the final resulting strain, which we called ‘SP’ (streptomycin-resistant *Pcc*), had an MIC that reached 50,000 mg/L; this was 2500 times higher than the 20 mg/L of the original parent strain. The RT-qPCR results showed that streptomycin binding site genes (*rsmG* and *rpsL*) were downregulated, while efflux pump genes (*acrD* and *mdtC*) were upregulated in SP (Figure S1). These results indicated that the SP strain is resistant to the antibiotic (streptomycin).

The MIC value of carvacrol was 200 μL/L for SP. We tested various combinations of carvacrol with streptomycin at different concentrations. As per Table 1, the most potent inhibition was at 100 μL/L and 6250 mg/L (FICI = 0.625), which resulted in a 2-fold and 8-fold reduction of carvacrol and streptomycin, respectively. This indicated that this particular combination exhibited significant antibacterial activity against SP.

### 2.2. Impact of Carvacrol, Streptomycin, and Their Combination on Conductivity and Movement

**Figure 1:**
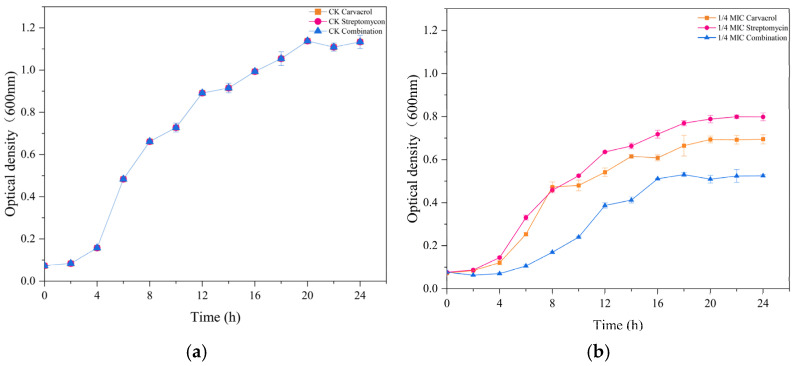

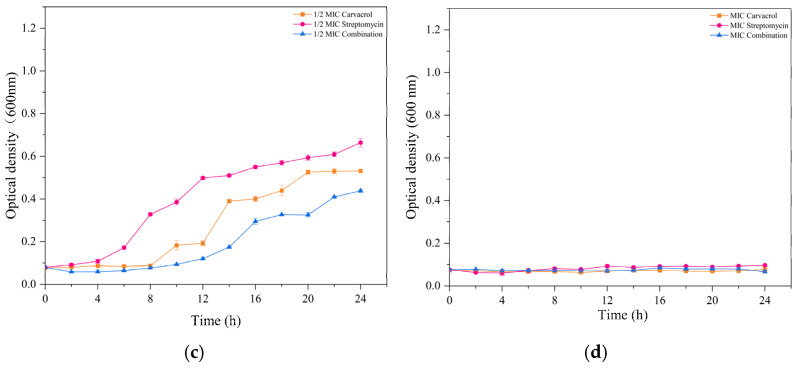
Growth dynamics of the SP strain were analyzed after treatment with different concentrations (ranging from 1/4 MIC to MIC) of carvacrol, streptomycin, and their combined application. (**a**) Untreated samples, and the sample groups treated with (**b**) 1/4 MIC, (**c**) 1/2 MIC, and (**d**) MIC concentrations of carvacrol, streptomycin, or both in combination.

The time–kill assay was conducted to assess to what extent carvacrol alone, streptomycin alone, and both in combination influenced the survival of SP cells. As Figure 1 shows, all treatments exhibited dose-dependent growth inhibition of SP, with the combination of MIC treatment having the strongest inhibitory effect. Moreover, carvacrol combined with streptomycin treatment evidently had the strongest inhibitory effect. These results indicated that all treatments were able to effectively suppress SP’s activity and growth, with the combination treatment having the greatest efficacy.

**Figure 2:**
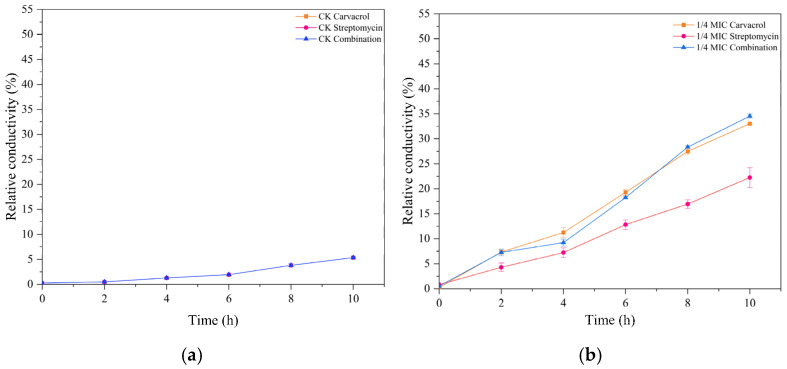

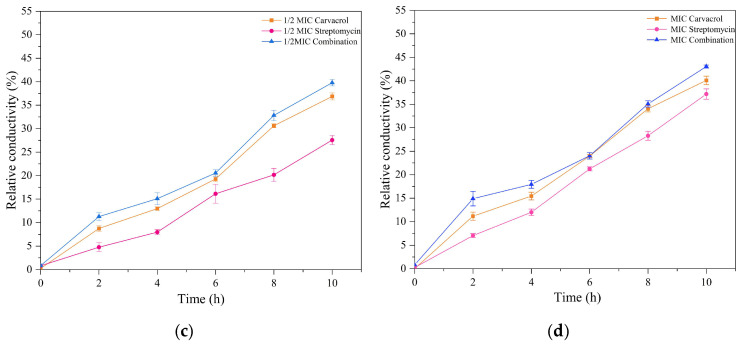
Changes in the relative conductivity of the SP strain were measured after treatment with different concentrations (ranging from 1/4 MIC to MIC) of carvacrol, streptomycin, and their combination. (**a**) Untreated samples, and the sample groups treated with (**b**) 1/4 MIC, (**c**) 1/2 MIC, and (**d**) MIC concentrations of carvacrol, streptomycin, or both in combination.

Figure 2 depicts the impact of carvacrol, streptomycin, and their combination on the membrane cell integrity of SP. Relative conductivity increased progressively with higher treatment concentrations and longer exposure times, reaching 40.09%, 37.18%, and 43.03% under the carvacrol, streptomycin, and combination treatments, respectively, after 10 h, compared to just 5.35% in the control. In addition, the relative conductivity of carvacrol combined with streptomycin was always greatest under different concentration treatments. Altogether, these results suggested that these treatments disrupted the permeability of SP’s cell membranes, leading to compromised cellular integrity.

**Figure 3:**
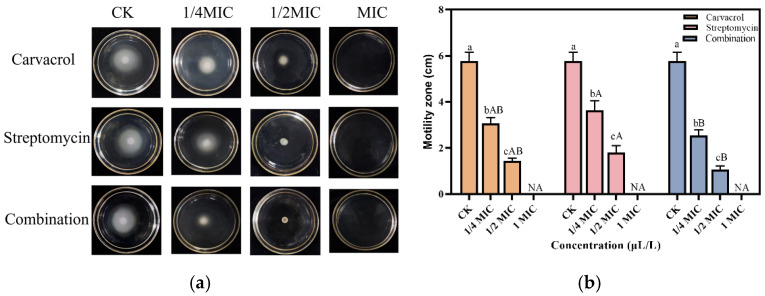
Changes in the motility of the SP strain were measured after treatment with different concentrations (ranging from 1/4 MIC to MIC) of carvacrol, streptomycin, and their combination. The untreated sample is the control group (CK). (**a**) Images of SP’s movement status in Petri dishes; (**b**) analysis of the measured SP movement diameters) (a–c: intra-group differences; A–B: inter-group differences).

The motility assays further supported these findings. After 24 h of incubation on a semi-solid medium containing 0.3% agar, SP’s diffusion diameters decreased with increasing drug concentrations (Figure 3). At 50% MIC, the diffusion diameters for the carvacrol, streptomycin, and combination treatments, respectively, were 0.75 cm, 0.81 cm, and 0.68 cm, with no observable diffusion at MIC. These results confirmed that all treatments significantly inhibited the motility of SP, with the combination treatment exhibiting the most pronounced effect.

### 2.3. Bacterial Morphology: SEM and CLSM Observations

**Figure 4:**
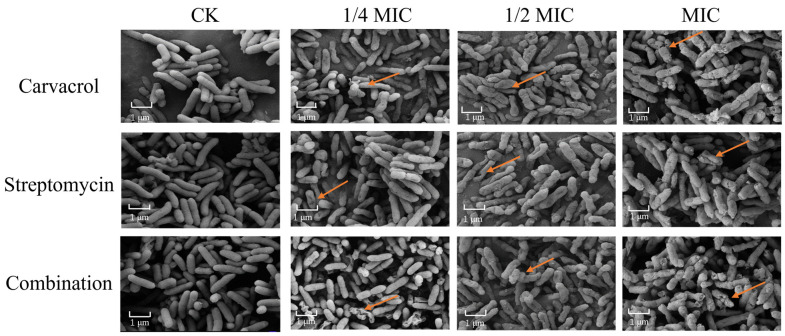
Changes in the structure of the SP strain were measured after treatment with different concentrations (ranging from 1/4 MIC to MIC) of carvacrol, streptomycin, and their combination. The untreated sample is the control group (CK).

The microstructural impact of carvacrol, streptomycin, and their combined application on SP cells was examined by SEM. Figure 4 shows their effects on cellular structural damage at different treatment concentrations. Evidently, when compared with the untreated samples, there were noticeable structural alterations, including the loss of smooth surfaces and a plump morphology, and even wrinkles or complete cracks. These observations suggested that applying carvacrol, streptomycin, or both in combination significantly compromises the structural integrity of SP cells, with higher concentrations causing more pronounced contractions and cellular damage.

**Figure 5:**
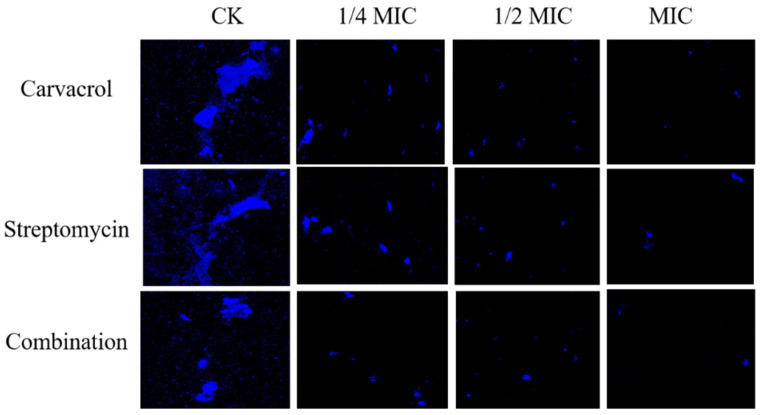
The effects of carvacrol, streptomycin, and their combinations applied at various concentrations (ranging from 1/4 MIC to MIC) on the altered membrane permeability of SP cells were investigated using CLSM. An untreated bacterial suspension served as the control group (CK).

Similar findings were obtained when using confocal laser scanning microscopy (CLSM). From Figure 5, it is evident that, relative to the control group, the intensity of the blue fluorescence emitted by bacterial cells clearly declines with increasing concentrations of carvacrol, streptomycin, and their combination. The magnitude of this reduction in fluorescence was particularly prominent in the combination treatment group. Hence, these observations suggested that carvacrol, streptomycin, or both in combination are capable of disrupting cell membrane integrity, thereby exerting bacteriostatic effects.

### 2.4. Effects of Carvacrol, Streptomycin, and Their Combination on the Extracellular Hydrolase Activity of SP

**Figure 6:**
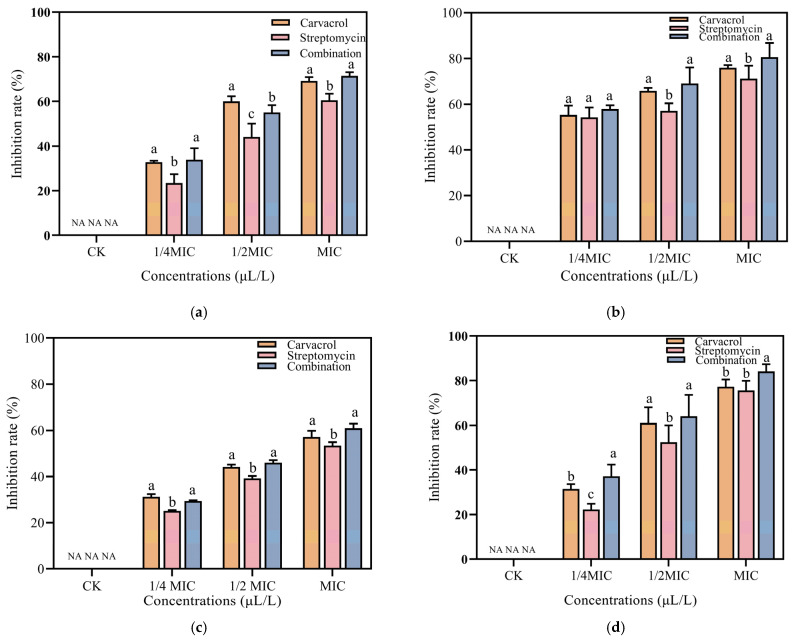
The effects of different concentrations of carvacrol, streptomycin, and their combination on the extracellular secretion of four enzymes: (**a**) protease, (**b**) pectinase, (**c**) polygalacturonase, and (**d**) cellulase (a–c: intra-group differences).

Pathogenic bacteria responsible for soft rot facilitate the decay of host plant tissue by producing extracellular enzymes, such as pectinase, polygalacturonase, cellulase, and protease, which contribute to the rapid progression of this plant disease [43]. As Figure 6 shows, treatment with carvacrol, streptomycin, or their combination markedly reduced the activity of these enzymes when compared to the untreated control group. Moreover, the inhibitory effect evidently intensified as the treatment concentrations increased, particularly under the combination treatment at the MIC concentration, for which the suppression of general enzymatic activity was most pronounced.

### 2.5. Evaluating Disease Control Effects in Different Vegetables

**Figure 7:**
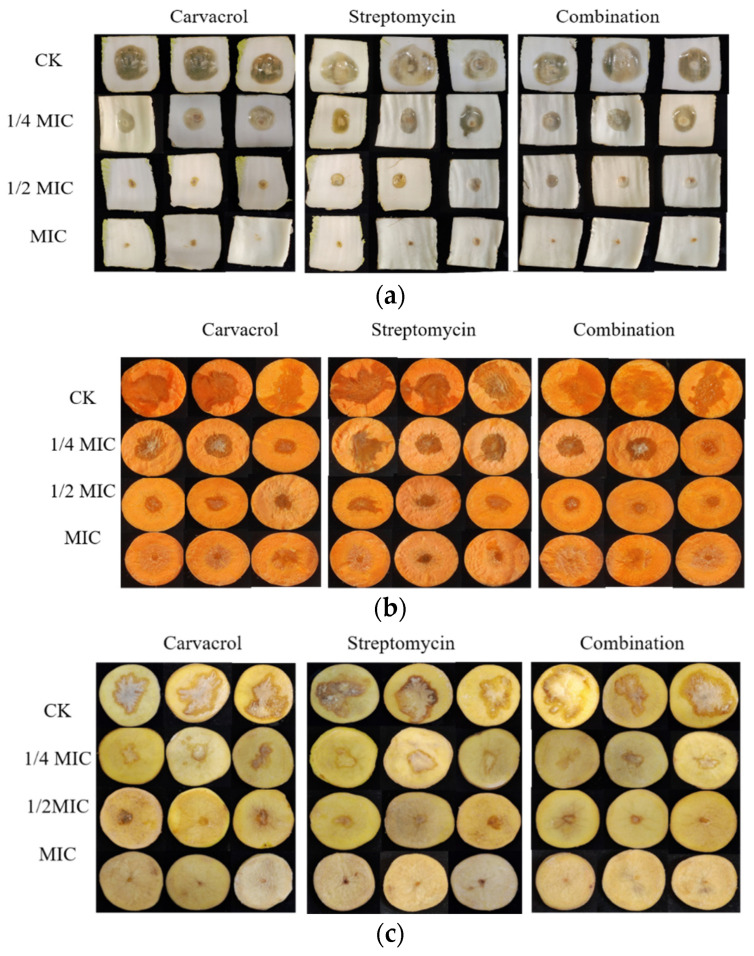
Therapeutic effects of carvacrol, streptomycin, and their combined application at different concentrations (ranging from 1/4 MIC to MIC) were evaluated in vegetables against soft rot. No chemicals were applied to the control group. (**a**) Chinese cabbage, (**b**) carrot, and (**c**) potato.

To assess the ability of carvacrol, streptomycin, or their combined use to mitigate soft rot disease caused by SP in Chinese cabbage, carrot, and potato, in vivo inoculation experiments were carried out. The highest MDR^x^, Rij^y^, and *p*ij scores were observed in the control groups of these three vegetables. In contrast, the lowest MDR^x^, Rij^y^, and *p*ij scores were recorded following their treatment with carvacrol, streptomycin, and both in combination (Table 1, Table 2 and Table 3). Furthermore, increasing concentrations of carvacrol, streptomycin, and their combination resulted in higher inhibition rates, with the inhibitory effect gradually becoming more pronounced (Figure 7). The effect of the carvacrol treatment on Chinese cabbages was on par with that of the combined treatment, while streptomycin treatment was less effective. For potato and carrot, the combined treatment had the best results, followed by carvacrol, with streptomycin alone being the least effective. Intriguingly, the combined treatment’s robust efficacy prevailed even when the carvacrol and streptomycin concentrations had fallen by 2- and 8-fold, respectively. This implied that the combined treatment harbored substantial potential for controlling soft rot.

**Table 1:** Assessment of the in vitro control efficacy of carvacrol, streptomycin, and their combined application for protecting Chinese cabbage samples against soft rot. Parameters include mean disease rating (MDR^x^), mean rank ($\bar{R}{\mathrm{ij}}^{y}$), and relative treatment effect ($\hat{p}\mathrm{ij}$) with 95% confidence intervals (CI) for disease severity. (a–c: intra-group difference).

Treatment		MDR^x^	$\bar{R}{\mathrm{ij}}^{y}$	$\hat{p}\mathrm{ij}$	Inhibition Rate (%)	95%CI for $\hat{p}\mathrm{ij}$	
						Lower	Upper
	CK	5.333	37.667	0.953	0	0.88	1.026
Carvacrol	1/4 MIC	3	24	0.603	37.92 ^c^	0.603	0.603
	1/2 MIC	1	7	0.167	71.75 ^b^	0.167	0.167
	MIC	1	7	0.167	91.00 ^a^	0.167	0.167
Streptomycin	1/4 MIC	3	24	0.603	27.88 ^c^	0.603	0.603
	1/2 MIC	2	16	0.397	58.74 ^b^	0.397	0.397
	MIC	1	7	0.167	86.54 ^a^	0.167	0.167
Combination	1/4 MIC	3	24	0.603	39.55 ^c^	0.603	0.603
	1/2 MIC	1.667	13	0.321	68.20 ^b^	0.01	0.65
	MIC	1	7	0.167	90.85 ^a^	0.167	0.167

**Table 2:** Assessment of the in vitro control efficacy of carvacrol, streptomycin, and their combined application for protecting carrot samples against soft rot. Parameters include mean disease rating (MDR^x^), mean rank ($\bar{R}{\mathrm{ij}}^{y}$), and relative treatment effect ($\hat{p}\mathrm{ij}$) with 95% confidence intervals (CI) for disease severity. (a–c: intra-group difference).

Treatment		MDR^x^	$\bar{R}{\mathrm{ij}}^{y}$	$\hat{p}\mathrm{ij}$	Inhibition Rate (%)	95%CI for $\hat{p}\mathrm{ij}$	
						Lower	Upper
CK	CK	9.333	38	0.962	0	0.917	1.003
Carvacrol	1/4 MIC	5	25.833	0.65	45.93 ^c^	0.212	1.081
	1/2 MIC	3.333	14.667	0.363	69.63 ^b^	0.134	0.593
	MIC	1.333	4.667	0.107	90.37 ^a^	−0.83	0.29
Streptomycin	1/4 MIC	5.333	28.167	0.709	42.96 ^c^	0.463	0.951
	1/2 MIC	3.333	14.667	0.363	65.93 ^b^	0.134	0.593
	MIC	2	8	0.192	83.70 ^a^	0.19	0.19
Combination	1/4 MIC	4	19	0.474	58.52 ^c^	0.47	0.47
	1/2 MIC	3.333	15.333	0.38	67.41 ^b^	−0.025	0.778
	MIC	1	3	0.064	92.22 ^a^	0.06	0.06

**Table 3:** Assessment of the in vitro control efficacy of carvacrol, streptomycin, and their combined application for protecting potato samples against soft rot. Parameters include mean disease rating (MDR^x^), mean rank ($\bar{R}{\mathrm{ij}}^{y}$), and relative treatment effect ($\hat{p}\mathrm{ij}$) with 95% confidence intervals (CI) for disease severity. (a–c: intra-group difference).

Treatment		MDR^x^	$\bar{R}{\mathrm{ij}}^{y}$	$\hat{p}\mathrm{ij}$	Inhibition Rate (%)	95%CI for $\hat{p}\mathrm{ij}$	
						Lower	Upper
CK	CK	5.333	36.333	0.919	0	0.773	1.035
Carvacrol	1/4 MIC	3	23	0.577	49.19 ^a^	0.577	0.577
	1/2 MIC	1.667	12	0.295	76.55 ^b^	−0.036	0.626
	MIC	1	6	0.141	91.53 ^a^	0.141	0.141
Streptomycin	1/4 MIC	3	23	0.577	47.56 ^c^	0.577	0.577
	1/2 MIC	2	15	0.372	71.66 ^b^	0.372	0.372
	MIC	1	6	0.141	88.93 ^a^	0.141	0.141
Combination	1/4 MIC	3	23	0.577	50.81 ^c^	0.577	0.577
	1/2 MIC	1.667	12	0.295	77.20 ^b^	−0.363	0.626
	MIC	1	6	0.141	92.18 ^a^	0.141	0.141

### 2.6. Transcriptomics Analysis

Transcriptional analyses were conducted using the RNA extracted from SP in the control group and the groups treated with carvacrol, streptomycin, and their combination. Table S1 shows that these results, overall, indicated minimal sequencing errors, and the high data quality and accuracy across all 12 samples supported their suitability and reliability for further analysis and processing.

**Figure 8:**
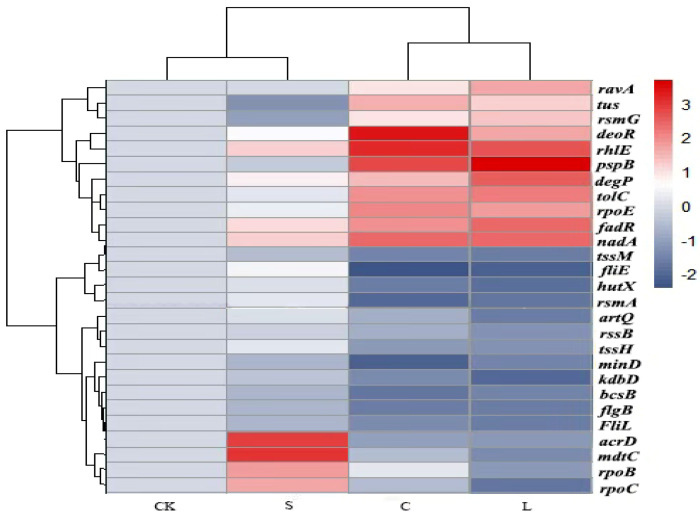
Heatmap showing the significant functional genes in the streptomycin–resistant *Pcc* strain (SP). CK: control group; S: treated exclusively with 1562 mg/L of streptomycin; C: treated with 100 μL/L; L: treated with a 1/2 MIC combination. (*p* < 0.05).

The transcriptome response of SP was visually summarized using a heatmap analysis (Figure 8). After the addition of streptomycin, the expression of genes related to the streptomycin binding site (*rsmG* and *rpsL*) was downregulated, while that of efflux pump-related genes (*acrD*, *mdtC*, *rpoB*, and *rpoC*) was upregulated; this showed that the SP strain could tolerate streptomycin. However, these genes exhibited opposite expression levels in the combined treatment group, which suggests that the combined treatment can notably reduce the activity of genes associated with resistance to streptomycin.

The expression levels of stress response- and outer membrane protein-related genes, including *rpoE*, *nadA*, *pspB*, *tolC*, and *fadR*, exhibited minimal changes in the streptomycin-treated group. In stark contrast, the expression of these genes markedly increased in the groups treated with carvacrol alone or in combination with streptomycin, which suggested that SP responds to external stimuli by regulating its coordinated expression of stress-related genes, thereby enabling these bacterial cells to quickly adjust to environmental changes. Additionally, genes associated with motility and biofilm formation, such as *flgB*, *fliE*, *fliI*, *hutX*, *tssM*, *bcsB*, and *artQ*, featured no notable expression changes in the streptomycin-treated group. However, their respective levels of expression were significantly downregulated in the carvacrol and combination treatment groups, indicating that the combination treatment suppressed the expression of genes associated with motility and biofilm development.

To sum up, the transcriptomic analysis revealed that carvacrol adversely impacts the pathogenic *Pcc* strain, SP, especially when applied in combination with streptomycin. This synergy between a phenol and an antibiotic arose by influencing key factors, such as the integrity of the cell membrane, bacterial movement, and the regulation of genes involved in bacterial inhibition.

### 2.7. Real-Time Quantitative PCR

**Figure 9:**
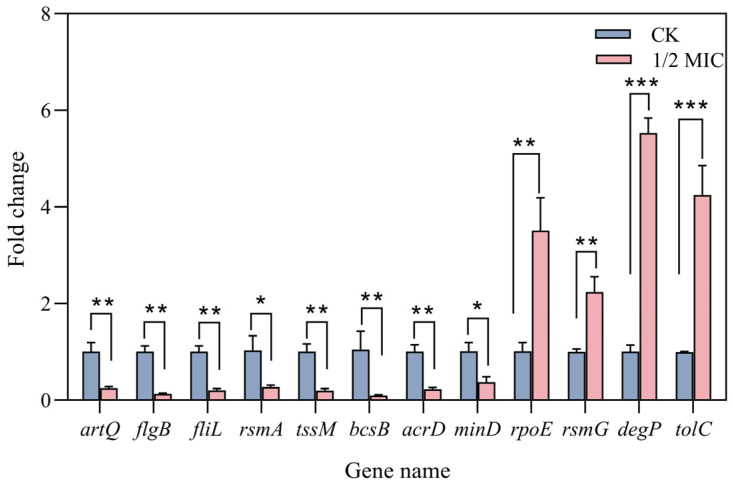
RT-qPCR verification of DEGs (differentially expressed genes) of SP in the control group (CK) and the group treated with a combination (1/2 MIC) of carvacrol and streptomycin. Data shown are the mean ± SD (n = 3), with significant differences indicated (*p* < 0.05). * *p* < 0.05, ** *p* < 0.01, *** *p* < 0.001.

To confirm the accuracy and dependability of the transcriptomic data, 13 DEGs were chosen for RT-qPCR validation based on the findings from the transcriptome analysis. These 13 DEGs consisted of five genes with higher expression levels and eight genes with lower expression levels: *rsmG*, *rpoE*, *tolC*, *pspB*, *degP*, *acrD*, *fliL*, *bcsB*, *tssM*, *flgB*, *minD*, *artQ*, and *rsmA*. As Figure 9 shows, for genes related to motility, biofilms, and the efflux pump, their expression was downregulated in SP. For genes related to streptomycin binding sites and the stress response, their expression was upregulated. These data and patterns corroborated the gene expression trends in the transcriptome data. Taken together, this suggested that carvacrol combined with streptomycin enhances the sensitivity of SP to the antibiotic streptomycin.

## 3. Discussion

Because *Pcc* is the main causal agent of soft rot, a highly destructive and widespread disease that afflicts plants, this bacterium is recognized as a major phytopathogen in agriculture [6]. Currently, the chief way to manage soft rot disease involves antibiotics and copper preparation treatments; for instance, streptomycin, one of the most widely used antibiotics in agriculture, is usually applied for soft rot control [11]. In addition to soft rot, streptomycin is also effective against other bacterial diseases, such as fire blight [44]. In the United States and Canada, streptomycin is approved for agricultural use under strict regulations to minimize the development of antibiotic resistance [45]. However, in most European Union countries, the use of streptomycin on crops is prohibited due to concerns over antimicrobial resistance and food safety [46]. This highlights the need to develop innovative and effective strategies to control soft rot while mitigating the risk of antibiotic resistance.

Natural compounds are recognized for their potent antibacterial properties and have been used globally to treat plant infections [47]. Carvacrol, an essential oil derived from Lamiaceae herbs such as oregano and thyme, acts as an efflux pump inhibitor that can enhance the susceptibility of resistant bacteria. It is has proven itself effective against methicillin-resistant strains [48] and has been applied to treat resistant *Salmonella enterica* in celery [49]. Additionally, carvacrol is capable of synergistic antibacterial effects with certain antibiotics, such as erythromycin and penicillin [50]. The present study reveals that carvacrol, especially when combined with streptomycin, is able to effectively inhibit SP, reducing both the MIC (minimum inhibitory concentration) and dosage requirements. In vitro experiments further show that this phenol–antibiotic combination can be used to successively treat soft rot caused by *Pcc*, either by forming a dense protective layer to prevent bacterial adhesion or by directly disrupting bacterial structures, thus limiting the pathogen’s ability to infect surrounding healthy plant tissue [51].

Although the applied combination of carvacrol and streptomycin clearly has significant bacteriostatic effects, the mechanisms by which they act upon SP remain unclear. Atomic force microscopy has shown that carvacrol’s antibacterial activity is largely driven by alterations to cell membrane permeability and cell viability [52]. It destabilizes lipid bilayers, which increases membrane permeability and leakage [53,54]. In our study, measurements of relative conductivity indicated that all treatments adversely affected the membrane integrity of *Pcc*, with the most pronounced change observed in the combined treatment, suggesting that the latter could achieve the greatest increase in permeability. In addition, DAPI staining, which binds to live cells and emits blue fluorescence, was used to assess membrane integrity. This method, which has been implemented by other researchers to examine membrane integrity, produced results similar to those of Li et al., who reported diminishing fluorescence intensity in response to increasing cyclic dipeptide concentrations in *S. aureus* [55]. We also found significant dose-dependent leakage of SP’s cell contents after applying the drug treatments. The follow-up SEM observations revealed the irreversible deformation of SP cells, including membrane wrinkling, rupture, and cytoplasmic leakage, all of which are consistent with the impacts observed by Huang et al. using dihydroartemisinin and cefuroxime against *E. coli* [56].

Antibiofilm agents are key to understanding antibacterial mechanisms, since biofilms function as natural barriers and modify the periplasmic microenvironment [57]. We know that SP relies on flagellar motility for biofilm colonization and host adhesion [58]. Here, the 0.5% agar motility assay showed that, with increasing concentrations of carvacrol, streptomycin, and their combined application, SP became less motile, indicating reduced biofilm formation. Additionally, carvacrol combined with streptomycin inhibited the virulence factors of this *Pcc* strain, which secretes enzymes such as polygalacturonase (Peh), pectate lyase (Pel), cellulase (Cel), and protease (Prt) that are responsible for soft rot symptoms in plant tissues. Extracellular hydrolase assays demonstrated that carvacrol, streptomycin, and their combination reduced Peh, Pel, Cel, and Prt production, with complete inhibition of pectate lyase at MIC concentrations; these findings are consistent with those of Zhang et al. [59].

We used transcriptome analysis to analyze the molecular mechanism(s) behind the combined action of carvacrol and streptomycin against SP. This analysis included treatments with carvacrol, streptomycin, and their combined application. The results revealed that treating SP with streptomycin alone increased its drug resistance, as indicated by downregulation of the ribosomal target genes *rpsL* and *rsmG*, along with upregulation of the efflux pump-related genes *acrD* and *mdtC*. However, the treatment combination of carvacrol and streptomycin weakens these resistance-related gene expression patterns. The *rpsL* gene encodes a ribosomal protein that serves as the predominant binding site for streptomycin at the conserved A site of 16S rRNA within the 30S ribosomal subunit, thereby inhibiting bacterial protein synthesis [60]. The *rsmG* gene encodes a methyltransferase responsible for modifying 16S rRNA via the 7-methylguanine modification; hence, deletion of *rsmG* disrupts this modification process, conferring streptomycin resistance [61]. Further, the *acrD*-encoded RND-type efflux pump and the *mdtC*-associated MdtABC system both contribute to drug resistance in bacteria by actively expelling streptomycin, reducing its intracellular concentration considerably [62]. Our findings suggest the tested carvacrol–streptomycin drug combination can mitigate streptomycin resistance in SP by reversing the expression of changed resistance-associated genes, thus bolstering this bacterial pathogen’s sensitivity to streptomycin.

Bacteria harbor evolved stress responses that enable them to adapt to various environmentally stressful conditions. In our study, the expression of stress-related genes (*pspB*, *degP*, *rpoE*, and *nadA*) was not significantly altered by the streptomycin treatment alone. However, the expression of these genes was significantly upregulated when carvacrol and streptomycin were used in combination. The gene *pspB* belongs to the phage shock protein (psp) operon; it is rapidly activated in response to membrane stress such as inner membrane damage and operates in coordination with other psp system genes to stabilize membrane structures and restore energy balance in the face of membrane damage [63,64]. The gene *degP* is strongly induced under oxidative stress and plays a critical role in degrading misfolded proteins [65]. Despite this essential role, this specific stress response demands substantial metabolic resources, which can inhibit bacterial growth to some extent. The synergy between oxidative stress and outer membrane repair is mediated by the σ^E^ factor encoded by the *rpoE* gene. As a key regulator of bacterial outer membrane stress, σ^E^ is activated when the outer membrane’s proteins are misfolded or when the membrane itself is damaged. By upregulating the expression of *degP* and other repair genes, σ^E^ enhances the tolerance of bacteria to outer membrane damage, but at the cost of greater resource allocation pressure on the cell [66]. Additionally, *nadA* encodes quinolinate synthase, which produces NAD, a critical molecule in organisms. In *Shigella* spp., *nadA* expression reduces bacterial virulence and prevents host cell invasion [67]. Collectively, these findings suggest that the differential expression of stress response genes induced by the combination of carvacrol and streptomycin strongly affects bacterial stress responses and survival. This underscores the potential of this drug combination for disrupting bacterial strains’ adaptive mechanisms and enhancing antibacterial efficacy.

Motility and biofilm-forming ability are both important criteria for assessing the effectiveness of antimicrobial agents against pathogenic bacteria. In this study, when SP was treated with streptomycin alone, there were no significant changes in its expression of motility- and biofilm-related genes, including *flgB*, *fliI*, *fliE*, *HutX*, *TssM*, and *BaeS*. However, their expression was significantly downregulated by the applied combination of carvacrol and streptomycin. Most bacteria possess a flagella system that aids their motility, consisting of a motor, hook, and filament connected by a rod-like structure [68]. Flagellar motility figures prominently in bacterial pathogenesis by facilitating critical processes, such as migration to, colonization of, and infection of hosts, as well as aiding survival within them and transmission to new hosts [69]. The formation and function of flagella are regulated by various genes, including *flgB*, *fliI*, and *fliE*. For instance, Jia et al. found that the upregulation of flagellar genes enhanced motility and virulence in *EHE O157*, which augmented its pathogenicity [70]. The *HutX* gene has been identified as being instrumental in biofilm formation in *Haemophilus parasuis*, playing a critical role in bacterial colonization [71]. Similarly, *TssM* regulates the functionality of the type VI secretion system, which is pivotal to bacterial population dynamics and their biofilm structures. Accordingly, the downregulation of *TssM* disrupts these processes, weakening the biofilm’s integrity [72]. Another gene, *BaeS*, encodes a sensing kinase in the BaeS/BaeR two-component regulatory system, which affects genes associated with motility and biofilm formation via signal transduction [73]. Therefore, although streptomycin treatment alone has limited inhibitory effects on the motility of SP and its biofilm formation, both of these activities are significantly reduced when applying carvacrol and streptomycin together. These experimental findings provide compelling evidence that carvacrol used in combination with streptomycin has strong potential as a biocontrol agent against SP.

This study has obtained promising outcomes in combating a streptomycin-resistant strain (SP). However, considerable work remains to be done to apply these results in practical SP control. Because bacterial resistance mechanisms to antibiotics vary among different strains [62,74], it is necessary to test a broader spectrum of resistant strains from diverse sources to validate the consistency and applicability of the findings. Based on the identified key genes involved in the response to the combined treatment, further functional studies, including gene knockout and complementation experiments, are imperative to elucidate the roles of these genes and associated pathways [75,76]. Considering that multiple environmental factors, such as temperature, humidity, and soil composition, can impact treatment efficacy [77], field trials are essential to confirm the practical feasibility and reliability of the combined treatment strategy under actual agricultural conditions [78].

Understanding the long-term impact of combination treatments on microbial communities and resistance evolution in agricultural ecosystems will provide valuable insights for developing sustainable disease management strategies.

## 4. Materials and Methods

### 4.1. Materials

Carvacrol (CAS 499-75-2) was purchased from Sigma-Aldrich (St. Louis, MO, USA), while streptomycin was bought from Shanghai Macklin Biochemical Co., Ltd. (Shanghai, China). Carvacrol and streptomycin were dissolved in dimethyl sulfoxide and pure water to prepare solutions of 100 μL/mL and 0.1 g/mL, respectively; these were used in the experiments described below. The original strain used in this study was *Pectobacterium carotovorum* subsp. *carotovorum* (*Pcc*), provided by the Institute of Plant Protection, Gansu Academy of Agricultural Sciences. These strains were incubated at 28 °C on LB medium (consisting of 5 g/L of yeast extract, 10 g/L of peptone, and 10 g/L of sodium chloride as key components). The vegetables consisted of potato (‘Kexin No. 4’), Chinese cabbage (‘Xibai No. 3’), and carrot (‘Xinheitian Wucun’) samples that were harvested from Lanzhou, Gansu, China. Only intact specimens, free from visible wounds or rot, were selected for use in this study. These vegetable samples were first disinfected using 1.0% sodium hypochlorite for 2 min, then thoroughly rinsed with sterile water and left to air-dry at 25 °C.

### 4.2. Culture of Resistant Strains and MIC of Carvacrol, Streptomycin, and Their Combination Against Resistant Strains

The SP strain was selected according to the methodology described by Zhou et al. [79]. First, *Pcc* was activated on LB culture medium and cultured for 16–24 h so it reached the logarithmic growth phase. The bacterial suspension was then inoculated into 100 mL of the LB culture medium containing streptomycin (10 mg/L) and incubated for 24 h. The culture was then successively moved onto a new medium, each with a higher streptomycin concentration (i.e., 20, 40, 80, 160, and 320 mg/L). This serial process was repeated until the bacteria grew at the highest concentration, ensuring stable resistance had been reached. The resulting *Pcc* strain, designated SP, was resistant to streptomycin.

The minimum inhibitory concentration (MIC) of carvacrol or streptomycin against SP was measured using the microdilution method [80]. Briefly, SP was cultured overnight in LB culture medium at 28 °C to attain the logarithmic growth phase. The bacterial suspension was then added to LB culture medium containing varying concentrations of carvacrol or streptomycin, or both (combination), and incubated at 28 °C for 24 h. The optical density (OD) at 600 nm of each bacterial suspension, before and after treatment with the different concentrations of carvacrol and/or streptomycin, was measured using a full spectrum microplate reader REAGEN LLC (Columbia, MD, USA). The MIC was determined as the minimum concentration of carvacrol or streptomycin where the change in OD at 600 nm was less than 0.1. Following the method of Singh et al. [81], the fractional inhibitory concentration index (FICI) of carvacrol and streptomycin was determined.

### 4.3. Time–Kill Assay

A time–kill assay was conducted to assess the impact of carvacrol, streptomycin, and their combination on SP by using the methodology described by Zhang et al. [82]. The SP strain was cultured overnight in LB medium at 28 °C until it reached the logarithmic phase. The bacterial suspension was inoculated into LB culture medium-complemented by various concentrations of carvacrol, streptomycin, or both together. Optical density (OD) at 600 nm was measured immediately after inoculation (0 h) and subsequently at 2 h intervals over a 24 h period. Carvacrol is often dissolved in solvents, and dimethyl sulfoxide (DMSO) is commonly used. To exclude the effect of the solvent 1% DMSO, we added DMSO to fresh Luria–Bertani (LB) solution as a control. The changes in OD values over time were used to infer bacterial growth dynamics.

### 4.4. Determination of Relative Conductivity

To determine the relative conductivity, we adopted the methodology outlined by Ju et al. [83]. First, SP was grown overnight in LB culture medium to the logarithmic growth phase. The bacterial cells then were collected by centrifugation at 5000 rpm for 10 min. Next, the conductivity of a pre-prepared 5% glucose solution was measured using a DDSJ-308A conductivity meter (Shanghai Yidian Scientific Instrument Co., Ltd., Shanghai, China). The bacterial pellet was then washed with this solution to create an isotonic suspension. This suspension was placed in hot water at 100 °C for 5 min and designated as the control group (L_0_). Different concentrations of carvacrol, streptomycin, and both in combination were incorporated into the glucose solution (L_1_). These treatments were applied to a bacterial suspension in the isotonic solution and incubated at 28 °C for 10 h, with conductivity measured every 2 h (L_2_). Each experiment was performed independently in triplicate. Relative conductivity was calculated as follows:

$$Relative\;conductivity\;(\%)=\frac{\left(L_{2}-L_{1}\right)}{L_{0}}\times 100$$

### 4.5. Motility Assay

Bacterial motility was assessed by the semi-solid plate method, as described in Fang et al. 2022 [84]. A culture medium comprising 0.5% agar, 1% tryptone, 0.5% yeast extract, and 1% sodium chloride was autoclaved and cooled to about 70 °C. Different concentrations of carvacrol, streptomycin, and their combination (1/4 MIC, 1/2 MIC, and MIC) were mixed in with the medium, while the control group consisted of LB with 1% DMSO. The center of each agar plate was inoculated with a 5 μL aliquot of the SP suspension for 24 h. Then, bacterial motility was quantified by measuring the size of the observed motility zone using ImageJ bundled with 64-bit Java 8 software. These experiments were performed in triplicate.

### 4.6. Bacterial Morphology: SEM and CLSM Analyses

The morphology of SP cells treated with carvacrol and streptomycin was examined by scanning electron microscopy (SEM) [85]. First, SP was cultured at 150 rpm and 28 °C until the logarithmic growth phase. Then, it was treated with carvacrol, streptomycin, or their combination at concentrations of 1/4 MIC, 1/2 MIC, and MIC for 14 h. The control group consisted of LB with 1% DMSO. Afterwards, the SP bacteria were harvested by centrifugation (at 5000 rpm for 10 min), washed with PBS, and preserved in 2.5% glutaraldehyde at 4 °C for 4 h. They were then dehydrated using a graded ethanol series (30%, 50%, 70%, 90%, and 100%) and vacuum freeze-dried for 24 h before undergoing gold plating. Their morphological alterations were examined under a scanning electron microscope (ZEISS GeminiSEM 500 (Oberkochen, Germany)). These experiments were performed in triplicate.

The CLSM analysis was performed using the method described in Cui et al. [51]. The SP strain was grown overnight at 28 °C until it reached the logarithmic phase; it was then inoculated into fresh LB culture medium, which contained different concentrations of carvacrol, streptomycin, or both. The control group consisted of LB with 1% DMSO. After 24 h, bacteria were centrifuged (8000 rpm, 10 min), washed with PBS, and stained with DAPI for 15–20 min in darkness. Finally, all samples were washed, placed on slides, and analyzed by CLSM (Olympus FV3000 (Tokyo, Japan)) at an excitation of 358 nm and an emission of 461 nm.

### 4.7. Effect of Carvacrol, Streptomycin, and Their Combination on the Extracellular Hydrolase Activity of SP

The plant cell wall consists of cellulose, pectin, polygalacturonic acid, and proteins. Corresponding extracellular hydrolases can degrade these components, leading to tissue softening and decay, which hastens disease development in plants. To evaluate the extracellular hydrolase activity, an agar plate assay was performed, where the diameter of the transparent region reflected the enzymatic level. For this experiment, the composition of the media used was as follows [47]: the cellulase medium contained 1% carboxymethyl cellulose; the pectinase medium contained 1% polygalacturonic acid, 1% yeast extract, 0.38 M CaCl_2_, and 100 mM Tris-HCl; the polygalacturonase medium contained 1% polygalacturonic acid, 1% yeast extract, 2.2 mM EDTA, and 110 mM sodium acetate; and the protein enzyme medium contained 10% skim milk powder. All of the above media each contained 0.8% agarose and 0.2% sodium azide.

### 4.8. Evaluating Control Effects on Chinese Cabbages, Carrots, and Potatoes

This study also investigated the effectiveness of carvacrol, streptomycin, and their combination on the growth of SP, ex vivo, by following the methodology outlined in Ji et al. [86]. Disease-free Chinese cabbage, carrot, and potato samples of uniform size were selected, surface-disinfected, and air-dried at room temperature under sterile conditions. For all treatment groups, a sterile syringe was used to inject 5 μL of the SP suspension (cultured to the logarithmic phase) into the center of each Chinese cabbage, carrot, or potato sample. These inoculated vegetables were left to air-dry for 3 h. Next, different concentration ranges of carvacrol, streptomycin, and their combination (spanning 1/4 MIC to MIC) were injected into the same wound site, with sterile water-treated vegetables serving as the controls. Sterile Petri dishes held the treated vegetables, which were covered with plastic and kept for 3 days in an incubator at 28 °C. Each sample was assessed for soft rot severity by measuring its lesion diameter to evaluate the treatments’ effectiveness at controlling soft rot disease in three vegetable types.

### 4.9. Transcriptomic Analysis

Given the complexity of their antibacterial mechanisms, transcriptomic sequencing was employed to investigate the molecular effects on SP from carvacrol combined with streptomycin, and to explore the enrichment of metabolic pathways and the changed expression levels of related genes [87]. To do that, four experimental groups were used for the transcriptomic analysis: a control group (CK), the 1/2 MIC carvacrol-treated group (C), the 1/4 MIC streptomycin-treated group (S), and the 1/4 MIC combination treatment group (L). Bacterial cells in these groups were sent to MAGIGENE (China) for total RNA extraction, library preparation, and sequencing. Each treatment was applied in triplicate.

### 4.10. Quantitative Real-Time PCR Validation

Quantitative real-time PCR (RT-qPCR) was conducted on the same RNA samples to validate the obtained transcriptome sequencing results [88]. Several differentially expressed genes (DEGs) identified in the transcriptome analysis were selected for validation. After normalization, the relative expression levels of these genes were quantified by applying the 2^−(ΔΔCT)^ approach. Following centrifugation, the bacterial cells were collected and washed with PBS, and the remaining liquid phase was removed. After extracting the total RNA, it was reverse-transcribed into cDNA using a commercially available reverse transcription kit. This cDNA was then amplified via qPCR on a StepOnePlus PCR system with the SYBR Green qPCR Master Mix. The genes’ corresponding primer sequences are provided in Table S2. Each reaction was analyzed in triplicate runs.

### 4.11. Statistical Analysis

All the experiments were conducted in triplicate, with their resulting data presented as the mean ± standard deviation (SD). Data were statistically analyzed in SPSS 26.0 software (IBM, Armonk, NY, USA), using one-way analysis of variance (ANOVA) followed by Duncan’s multiple range test. A *p*-value below 0.05 was considered significant.

## 5. Conclusions

We have shown that using carvacrol combined with streptomycin exhibits strong antibacterial activity against SP by disrupting biofilm permeability and inhibiting both motility and enzyme activity. The MIC value for SP (streptomycin-resistant *Pectobacterium carotovorum* subsp. *carotovorum*) was significantly lower for the combined treatment than either treatment using carvacrol or streptomycin alone. The bacterial transcriptome results show that carvacrol markedly suppresses the expression of drug resistance genes, such as *rsmG*, *rpsL*, *acrD*, and *mdtC*, suggesting that carvacrol is able to restore the sensitivity of SP to streptomycin, thereby enhancing the latter’s antibacterial efficacy. Overall, these findings emphasize the potential of combining carvacrol with streptomycin as an effective control strategy against soft rot disease in crops, offering a promising approach for using natural compounds in tandem with antibiotics to combat streptomycin-resistant pathogens.

## Data Availability

Data are contained within the article.

## Supplementary Materials

The following supporting information can be downloaded at: https://www.mdpi.com/article/10.3390/plants14060908/s1, Figure S1. Differential expression of streptomycin tolerance genes in *Pcc* and SP; Table S1: Primers used for real-time quantitative PCR analysis. Table S2: Quality control of transcriptome sequencing data.
