# Supplementary material for: Carvacrol and Streptomycin in Combination Weaken Streptomycin Resistance in Pectobacterium carotovorum subsp. carotovorum
Source: Plants (Basel). 2025 Mar 14;14(6):908. doi: 10.3390/plants14060908 (PMC11944609; doi:10.3390/plants14060908)
Supplement: Supplementary file 1 [file plants-14-00908-s001.zip › plants-3471055-supplementary.pdf]

Table S1. Primers used for real-time quantitative PCR analysis.

Gene	Forward Primer(5'−3')	Reverse Primer (5'−3')
rsmG	TTGAGCCACATTTACACGGACAG	TGCGACTCAGGGCGAACG
rpoE	CATGGCTGTATCGTATCGCAGTG	GGCATCAATGTCGCTGGAAGG
tolC	CCGCAACCTGAACCTGTTATCC	TCCAGCGTCGGCATGTAGC
pspB	GCACCGATATGGCTTTGGCTAC	CGCATACGATTGGACTCTTCCG
degP	GGTGCGTTTGTAAGCCAGGTG	GCTGCTGATCGCCTTACCATTC
acrD	AGAACAGTCAGGTGTCGGTAGG	GCTGCGGCGTCTCTAACAAC
fliL	GGCACCCGCAGCACCAG	GCAAGTTCGACCAGGACGTAAAC
bcsB	ACGGCGGTAAGCAGTTCATTATC	ACGGACCAACGGTGTCTATCATC
tssM	CTCGCTGTTCTCGTTCGTTCG	ATCCTCGCCTGTCGCCAAC
flgB	GGATGGCAACACGGTCGATATG	TCAGAACGGTCAGGCTGGATTG
minD	GCTGGTTGGCGTGATTCCG	GGCTTCCGCATCCAGAATGAC
artQ	CGCTGCTGTATGCTGCTTACG	ACCCGATTCCCATTGCCCTTG
rsmA	AGGGTGAATCGCCGAAACAATG	CAAGGTCACTTCGCCCGTAAAC

Table S2 Quality control of transcriptome sequencing data

Sample	Raw reads	Clean reads	Q30(%)	GC(%)	Clean Reads(%)	Total mapped(%)
CK.1	16630360	16303390	96.68	48.92	98.03	63.57
CK.2	16038968	15720732	96.62	50.13	98.02	74.06
CK.3	15647280	15302752	96.49	48.98	97.8	61.31
C.1	14406172	13647604	94.88	50.95	94.73	93.47
C.2	14323110	13220514	95.48	50.75	92.3	91.88
C.3	15174508	14798116	94.72	51.26	97.52	92.48
S.1	14821410	14481232	96.41	52.06	97.7	96.64
S.2	15403750	15094758	96.42	51.97	97.99	96.15
S.3	14403658	14120274	96.14	51.98	98.03	96.31
L.1	14427136	14141652	95.5	51.17	98.02	95.16
L.2	16860440	16396726	94.56	51.07	97.12	92.64
L.3	17374190	17044380	93.72	51.48	98.1	96.45

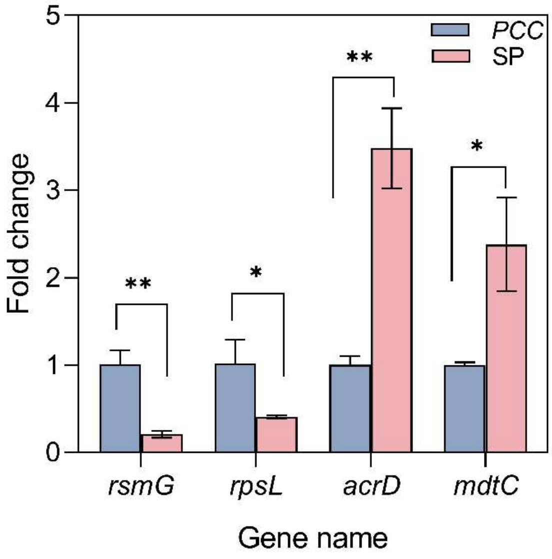

Figure S1 Differential expression of streptomycin tolerance genes in *Pcc* and SP
